# Using the electronic health record to provide audit and feedback in medical student clerkships

**DOI:** 10.1093/jamiaopen/ooae090

**Published:** 2024-09-23

**Authors:** Jacqueline Xu, Matthew A Silver, Jung Kim, Lindsay Mazotti

**Affiliations:** Department of Clinical Science, Kaiser Permanente Bernard J. Tyson School of Medicine, Pasadena, CA 91101, United States; Department of Clinical Science, Kaiser Permanente Bernard J. Tyson School of Medicine, Pasadena, CA 91101, United States; Southern California Permanente Medical Group, San Diego, CA 92123, United States; Department of Health Systems Science, Kaiser Permanente Bernard J. Tyson School of Medicine, Pasadena, CA 91101, United States; Ronald O. Perelman Department of Emergency Medicine and Institute for Innovations in Medical Education, New York University Grossman School of Medicine, New York, NY 10016, United States; Sutter Health, Sacramento, CA 95833, United States

**Keywords:** undergraduate medical education, clinical clerkships, electronic health record, formative feedback, practice-based learning and improvement

## Abstract

**Objectives:**

This article focuses on the role of the electronic health record (EHR) to generate meaningful formative feedback for medical students in the clinical setting. Despite the scores of clinical data housed within the EHR, medical educators have only just begun to tap into this data to enhance student learning. Literature to-date has focused almost exclusively on resident education.

**Materials and Methods:**

Development of EHR auto-logging and triggered notifications are discussed as specific use cases in providing enhanced feedback for medical students.

**Results:**

By incorporating predictive and prescriptive analytics into the EHR, there is an opportunity to create powerful educational tools which may also support general clinical activity.

**Discussion:**

This article explores the possibilities of EHR as an educational resource. This serves as a call to action for educators and technology developers to work together on creating health record user-centric tools, acknowledging the ongoing work done to improve student-level attribution to patients.

**Conclusion:**

EHR analytics and tools present a novel approach to enhancing clinical clerkship education for medical students.

## Introduction

For medical students, constructive feedback is critical for a developing physician, whether it is a hallway conversation with attending supervisors or scores from clinical simulation-based exams. Yet, disparate feedback sources and assessments subject to biases and incomplete evidence from the clerkship experience leave many medical students fearful of subjective, and presumptively ineffectual, clerkship grading.^1^^,^^2^ While the value of subjective assessment should not be undermined, medical students and educators alike acknowledge the need for more structured, objective benchmarks, especially in the context of continued clerkship grade inflation and racial & ethnic bias.^3^^,^^4^

One opportunity for such objectivity lies within the electronic health record (EHR). The EHR is now a ubiquitous part of clinical care and, therefore, part of medical education. The EHR offers the potential to structure audits and feedback for students, generating data on patient volume, patient outcomes, and panel-level population health.^5^ Sebok-Syer et al^6^ featured the EHR not only as a *measuring stick* for trainee competency development, but an *educational tool itself—providing valuable feedback data for learners*. The authors highlighted the importance of maximizing the EHR as a source of timely, individualized feedback with performance metrics adapted to resident physicians operating in a team-based setting. This article reviews how the EHR can be expanded in undergraduate medical education (UME) to enhance clerkship learning and student development.

## Generating objective benchmarks to monitor clinical learning

EHR-based medical education research shows the potential to generate helpful feedback by identifying training gaps. Rajkomar et al^7^ found, via EHR note analysis, that only 3% of all first-year residents on an internal medicine service had exposure to the top 10 ICD-9 diagnoses within their institution. Yet, medical students can often “choose” which patients to see in clinic and which surgeries to participate in. However, the pressure to perform and desire to excel may lure students into selecting patients they already feel confident and competent in managing. Additionally, students sometimes lack awareness of their knowledge gaps and may feel uncertain about the areas they need to address before entering residency. Clerkship directors often have their own expectations with regards to common patient cases each student should have exposure. Enabling student documentation and EHR interaction serves as a valuable data source for educators to track and assess activities in the clinical learning environment. It also offers an opportunity to align clinical course expectations with the dynamic and multifaceted realities of the clinical setting.

One existing but imperfect method of tracking student clinical learning is the “required clinical experiences (RCE)” log. To standardize and ensure comparability across all student clerkship experiences, accreditors require each institution to generate a list of patient diagnoses to which students manually self-report exposure. At our institution, for example, students must report at least one exposure to a patient seeking contraceptive counseling, an adult with congestive heart failure, management of a patient with a personality disorder, and so on. While some RCEs are encountered frequently (eg, children with upper respiratory infection symptoms), others may be encountered only once (eg, children with growth delay). The frequency of RCE exposure is influenced by clinical prevalence and student choice. Instead of requiring students to manually track RCEs and saturate the learning experience with accreditation reporting, existing EHR-based tools could instead automate and track encounters where students were meaningfully involved in patient care. Future iterations of these tools could integrate predictive or prescriptive analytics that identify gaps in patient presentations before residency. This approach encourages students to broaden their experience rather than focus solely on performance (Table 1).

**Table 1.** Example list of an EHR augmented required clinical experiences (RCEs) for a core clerkship medical student.

Specialty	**RCE** (*non-exhaustive*)	Example of EHR enhancement strategy
Internal Medicine/Family Medicine (outpatient)	Evaluate a patient presenting with each of the following: • Headache • Joint pain • Dyslipidemia	Proactively highlight scheduled appointments for which the chief concern aligns to RCE
Internal Medicine/Family Medicine	Manage a patient with each of the following: • Congestive heart failure • Coronary artery disease • Solid tumor cancer • Urinary tract infection	Auto-log student encounters for which the primary diagnosis aligns to RCE
Obstetrics & Gynecology	Assist in each of the following procedures: • Pap smear • Spontaneous vaginal delivery • Caesarian section • Gynecologic surgery	Record all procedures performed during designated shift, allowing student to mark those they assisted vs observed
Pediatrics	Evaluate a child with growth concerns, including constitutional delay, microcephaly, obesity, short stature, and others	Flag patients with abnormal growth curves results for possible student involvement
Psychiatry	Perform a suicide assessment	Track patients with EHR-based suicide assessment completed by student
Surgery	Assist in each of the following procedures: • Inguinal hernia repair • Bowel surgery • Cholecystectomy	Track surgeries students were involved in by operative notes authored or orders placed/pended, then create student facing dashboards that display operative details (eg, open vs laparoscopic vs robotic) to highlight surgical methods they have yet to experience.

## Tracking patient outcomes to further professional development

Allowing students to track clinical outcomes on patients they have seen is another key but often missing piece in the medical student clerkship experience. Brisson et al^8^ reported on the medical student practice of independently tracking former patients in the EHR, without institutional direction. Ninety-three percent of students reported benefits to this tracking, allowing them to follow-up on pending studies and confirm a diagnosis or follow progress during treatment. Forty percent of student’s chart-checked patients, out of curiosity for their clinical outcomes. Learning about a patient’s outcome from the EHR is critical to understanding disease prognoses and can be a humbling reminder of the challenges inherent in the medical profession. How many patient outcomes—readmissions, emergency department visits, deaths, surgeries, sequelae, and other complications—were missed and not shared back to the medical student? Attendings and residents are more involved in patient care and may receive regular updates from other members of the healthcare team or, in some cases, automatic EHR notifications about patient deaths or rehospitalizations, but medical students are often left in the dark. Yet, understanding ultimate patient outcomes and the “big picture” view of a patient’s health is perhaps one of the most integral pieces of a clinical education. Medical students should know what happens to the patients they see. Medical students need to see the entire story of an illness, beyond single snapshot presentations and into an entire arc of detection, diagnosis, and management. Diagnosing the young, asymptomatic patient with diabetes is a different story after students have cared for diabetic patients after lower extremity amputations. Educators and developers could leverage EHR-based patient lists to provide learners with key updates on patient outcomes. The same way residents and attendings may receive automated-EHR notifications (eg, in-basket messages) for patient deaths or rehospitalizations on their panel, medical students could receive similar notifications via manually curated patient lists. For example, a patient seen by a medical student in the intensive care unit could be marked to follow, with future updates about the patient’s disposition shared via EHR-based messages. Such a system could also be leveraged for practicing inpatient clinicians without panels, who may desire more information about their longitudinal clinical care outcomes. While our motives in the UME space are primarily educational, these potential EHR-based tools have clear clinical benefits as well.

## Building into the “panel management” view

As our healthcare system shifts toward ambulatory care, medical student clerkship training is becoming increasingly outpatient-based.^9^ Graduate medical education expected competencies include “Practice-Based Learning and Improvement,” requiring residents to monitor and improve outcomes of larger swaths of patients. To that end, medical students, our future residents, will need exposure to this panel view that has a greater emphasis on patient outcomes. Of course, given the limitations in clinical care delivered by a medical student, EHR-based measures of performance cannot be used for trainee assessment without certain considerations.^10^^,^^11^ Lower overall patient volumes and greater dependence on team-based care present the challenge of defining individually attributable metrics at the student level. However, these should not preclude educators from removing this critical piece of clinical experience from the clerkship altogether, and studies are already underway to support individual attribution at the graduate medical education level. In particular, the concept of TRainee Attributable & Automatable Care Evaluations in Real-Time (TRACERs) proposed by Burk-Rafel et al^12^ could be married with EHR-based tools to enhance trainee learning and feedback. Medical student notes or pended orders could be assessed with the TRACERs framework. For example, educators may assess to what extent student notes document consideration of all 4 medication classes in guideline-directed medical therapy for patients with congestive heart failure (whether prescribed or not, as each clinical scenario may differ based on patient comorbidities or clinical status). Another example is whether medical student notes outline necessity of head imaging for patients presenting with headaches, based on presenting clinical risk factors. These examples build upon Table 1 sample of RCE, and future TRACERs could support each institution’s RCEs to enable deeper, quantitative views of medical student learning and patient outcomes.

## Conclusion

Optimizing and leveraging the EHR is one example of an “on-the-job” training tool and feedback loop that we are underutilizing in medical education. Our arsenal of technological and artificial intelligence decision-making tools within healthcare is expanding—from digesting note content with natural language processing to synthesizing clinical evidence with ChatGPT-like virtual assistants. The potential for students to grow into self-directed adult learners, without completely relying on individual supervisors, becomes a reality. However, we risk leaving our profession behind with blunt tools. This has led our team to begin designing EHR-based tools for medical students to monitor their own clinical experiences and learning, the start of “precision medical education” as described by Triola and Burke-Rafel.^13^^,^^14^ EHR-based tools allow us to empower students in becoming an active participant of their professional development, creating agency for learners to invest in our own journeys with clearer patient outcomes.

## Data Availability

No new data were generated or analyzed in support of this research.
